# Dry needling in a multimodal rehabilitation protocol following rotator cuff repair surgery: study protocol for a double-blinded randomized sham-controlled trial

**DOI:** 10.1186/s12891-023-06269-1

**Published:** 2023-04-26

**Authors:** Faeze Naseri, Mehdi Dadgoo, Mohammadreza Pourahmadi, Morteza Nakhaei Amroodi, Shirin Azizi, Pouria Tabrizian, Ali Amiri

**Affiliations:** 1 Department of Physiotherapy, Rehabilitation Research Center, School of Rehabilitation Sciences, Iran University of Medical Sciences, Madadkaran All., Shahnazari St., Madar Sq., Mirdamad Blvd., Tehran, Iran; 2 Department of Orthopedics, Bone and Joint Reconstruction Research Center, School of Medicine, Iran University of Medical Sciences, Tehran, Iran

**Keywords:** Dry needling, Shoulder pain, Trigger point, Rotator cuff repair, Rehabilitation

## Abstract

**Background:**

Rotator cuff tear (RCT) is one of the main causes of shoulder pain and dysfunction. Rotator cuff repair (RCR) is a common surgical procedure for the management of RCTs. Presence of myofascial trigger points (MTrP) as a result of surgical procedure can aggravate postoperative shoulder pain. The purpose of this protocol is to describe a randomized controlled trial design to evaluate the effect of implementing 4 sessions of myofascial trigger point dry needling (MTrP-DN) within a multimodal rehabilitation protocol following RCR surgery.

**Methods:**

Forty-six participants aged 40–75 will be recruited having postoperative shoulder pain after RCR and meeting the inclusion criteria. Participants will be randomly divided into 2 groups: One group will undergo MTrP-DN, manual therapy, exercise therapy and electrotherapy and the other will receive sham dry needling (S-DN), manual therapy, exercise therapy and electrotherapy. This protocol will cover 4 weeks of intervention. The primary outcome measure will be the Numeric Pain Rating Scale (NPRS) for pain. Secondary outcome measures will be Shoulder Pain and Disability Index (SPDI), range of motion (ROM), strength and adverse events.

**Discussion:**

This is the first study to investigate the use of 4 sessions of MTrP-DN in combination with a multimodal rehabilitation protocol for postoperative shoulder pain, restriction, weakness and dysfunction following RCR. The results of this study may help to determine the effect of MTrP-DN on various outcomes after RCR surgery.

**Trial registration:**

This trial was registered at the (https://www.irct.ir), (IRCT20211005052677N1) on 19/2/2022.

## Administrative information

The numbers in curly brackets comply with the numbered items in the SPIRIT checklist 2013 [1].

**Table:** 

Title {1}	Dry needling in a multimodal rehabilitation protocol following rotator cuff repair surgery: study protocol for a double-blinded randomized sham-controlled trial.
Trial registration {2a, 2b}	IRCT20211005052677N1 (IRCThttps://www.irct.ir) [registered on 19 February 2022]
Protocol version {3}	1st protocol version, October, 2022.
Funding {4}	Iran University of Medical Sciences will support the present study but the authors declare that they did not receive any funding sources.
Protocol contributors’ details {5a}	Faeze Naseri, PT, BSc^1^; Mehdi Dadgoo, PT, PhD^1^*; Mohammadreza Pourahmadi, PT, PhD^1^; Morteza Nakhaei Amroodi, MD^2^; Shirin Azizi, BSc^1^; Pouria Tabrizian, MD2; Ali Amiri, PT, PhD^1^. ^1^Rehabilitation Research Center, Department of Physiotherapy, School of Rehabilitation Sciences, Iran University of Medical Sciences, Tehran, Iran. ^2^Bone and Joint Reconstruction Research Center, Department of Orthopedics, School of Medicine, Iran University of Medical Sciences, Tehran, Iran.
Name and contact information for the trial sponsor {5b}	This study has no funding sources.
Role of study sponsor and funder {5c}	This study will be implemented under the supervision of School of Rehabilitation Sciences, Iran University of Medical Sciences, Tehran, Iran. Although the mentioned center has no role in the study design, data collection, data analysis, data management, data interpretation, and the dissemination of the study results. This study has no funding sources.
Data monitoring committee {5d}	Faculty members of the Department of Physiotherapy, School of Rehabilitation, Iran University of Medical Sciences are responsible for overseeing the trial.

## Background {6a}

Shoulder pain is the third common musculoskeletal disorder with a prevalence of 30% in lifetime [2]. Rotator cuff tear (RCT) following trauma or degenerative changes is one of the most common causes of shoulder pain and dysfunction, which accounts for 20% of shoulder injuries [3]. The risk of RCT increases in the aging population. As a result, 25.6% of people up to the age of 60 suffer from tearing of these muscles. RCT increases up to 50% in people who are in their 80s [4]. Pain, range of motion (ROM) restriction, and weakness during arm elevation are the common complaints of patients with RCT which could lead to loss of upper extremity function [5, 6].

Rotator cuff repair (RCR) is recommended for patients with partial-thickness tears whose symptoms have not changed after 3 to 6 months of non-operative treatments or patients with symptomatic full-thickness RCTs [7, 8]. Additionally, in a 10-year follow up study, RCR procedure has shown better results in outcomes compared to non-operative treatments for tendon tears [9]. RCR incidence has increased by 238% over the past decades, ranging from 23.5 to 83.1 cases per 100,000 people [4].

An appropriate rehabilitation protocol after RCR surgery plays an important role in restoration of upper extremity function [10]. The main goals of the rehabilitation program for patients with RCR include pain relief, recovery of passive range of motion (P-ROM) and active range of motion (A-ROM), improving the strength of shoulder girdle muscles, avoiding stiffness and muscle atrophy, and returning to activities of daily living [10]. Recent studies have shown that physiotherapy regimens including manual therapy and electrotherapy combined with exercise programs are beneficial for patients with rotator cuff disease [11].

Surgical procedure as a mechanical trauma to the soft tissues surrounding the surgical incision can be a potential mechanism for activation of myofascial trigger points (MTrP) [12]. MTrPs are the hyperirritable spots in the taut band of skeletal muscles. Active MTrPs cause spontaneous pain and elicit referred pain, but latent MTrPs reproduce none of the symptoms for the patients. The activation of MTrPs may aggravate postoperative shoulder pain and disrupt function of the upper extremity [13]. As a result, management of active MTrPs can be beneficial for patients following RCR [14].

Dry needling is an invasive treatment for MTrP relief where an acupuncture needle is inserted in the muscles. Myofascial trigger point dry needling (MTrP-DN) in combination with other physiotherapy interventions is widely used by physiotherapists around the world [15]. Recent studies have provided evidence for using dry needling for patients with MTrPs of the neck and upper extremity [16]. However, the effectiveness of MTrP-DN in postoperative patients is debatable [14]. A recent randomized clinical trial by Arias-Buría et al. found that including a single session of trigger point dry needling in a multimodal physiotherapy approach may improve the outcomes for patients with postoperative shoulder pain [14]. Although another randomized clinical trial by Halle et al. found that shoulder girdle muscles’ dry needling along with physiotherapy program after shoulder stabilization surgery did not significantly improve the outcomes in comparison with physiotherapy program alone [17]. Consequently, this study aims to determine the effects of MTrP-DN combined with a multimodal rehabilitation protocol on pain, glenohumeral ROM, shoulder girdle muscles’ strength, and Shoulder Pain and Disability Index (SPDI) after RCR.

### Objectives and hypothesis {7}

The primary objective of this trial is to determine the effect of MTrP-DN combined with a multimodal rehabilitation protocol compared to S-DN combined with a multimodal rehabilitation protocol on pain for patients following RCR. The secondary research objectives are to determine the effects of MTrP-DN compared to S-DN combined with a multimodal rehabilitation protocol in both groups on ROM, shoulder muscles’ strength, and SPDI. The hypothesis is that those participants who receive MTrP-DN in their rehabilitation program would exhibit greater improvements in pain, ROM, shoulder muscles’ strength, and SPDI. The null hypothesis is that those participants who receive MTrP-DN will not significantly differ from the participants who will receive S-DN in the mentioned outcomes.

## Methods

### Trial design {8}

**Fig. 1:**
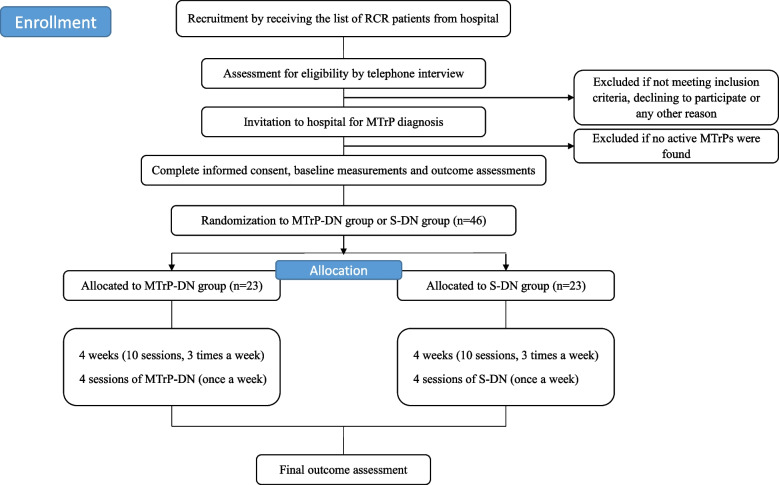
Consort flowchart related to the stages of the study

Study protocol for a single center, superiority, randomized, double-blinded, sham, controlled trial with a parallel group of 46 patients. The allocation ratio will be 1:1. This study complies with the recommendations of the SPIRIT 2013 statement. A CONSORT flowchart related to the stages of the study is shown in Fig. 1.

### Study setting {9}

This study will be conducted in the physiotherapy clinic of Shafa Yahyaian Hospital, Tehran, Iran. The participants of this study are the patients who have undergone RCR surgery in Shafa Yahyaian Hospital by an expert shoulder and elbow surgeon at this center (MNA).

### Participants {10}

The participants scheduled for open RCR due to a RCT will be recruited in this trial. The open RCR consists of a 5-cm incision using sabercut approach and detachment of deltoid muscle to reattach the rotator cuff tendons to greater tuberosity with transosseuos sutures [10]. The size of the tendon tear is different in each patient and all operations will be performed by the expert surgeon of the Shafa Yahyaian Hospital (MNA) who has more than 15 years of experience in shoulder and elbow surgery using the same procedure for the RCT patients. The inclusion and exclusion criteria can be found in Table 1.

**Table 1:** Inclusion and exclusion criteria

Inclusion criteria	Exclusion criteria
1) Status post RCR surgery 2) Aged between 40 and 75 years 3) Experiencing shoulder pain after 5 weeks of surgery 4) Presence of active MTrPs in shoulder girdle muscles’ palpation	1) Phobia of needle 2) History of coagulation disorders and intake of anticoagulants 3) History of head and neck surgery 4) Radiculopathy and myelopathy disorders 5) Pregnancy, 6) No active MTrPs were found

### Assessor and therapist

The patients will be assessed by a blind assessor (SA) who is a physiotherapist with more than 3 years of clinical experience. The patients will be treated by a certified clinical physiotherapist (FN) with more than 4 years of experience in MTrP-DN and the treatment of patients following RCR surgery.

### Intervention {11a}

#### Multimodal rehabilitation protocol

Each treatment session will be divided into 3 parts:

The first part will be devoted to electrotherapy, during which the participants in both groups will receive conventional Transcutaneous Electrical Nerve Stimulation (TENS) on the afflicted shoulder at a frequency of 120 Hz and a duration of 50 μs for 20 minutes [18].

The second part will be devoted to manual physiotherapy. Passive joint mobilization techniques used in previous studies will be implemented in this study as well [14, 19–21]. Glenohumeral and scapulothoracic joints mobilization techniques are chosen for this trial [22]. Distraction, inferior glide, posterior glide and anterior glide will be applied for glenohumeral joint mobilization for 2 sets of 20 repetitions [23]. Inferior glide, superior glide, medial glide, lateral glide, upward rotation, downward rotation, depression and retraction will be applied for scapulothoracic joint mobilization with 10 repetitions of each movement [23].

The third part will be devoted to therapeutic exercises. Exercise progressions will be implemented based on the protocols presented by Giangarra et al. The exercise program in this part will include ROM and strengthening exercises, which will progress over the course of 4 weeks. The first three sessions will focus on P-ROM exercises (first and second week), followed by active assistive range of motion (AA-ROM) exercises from the fourth session (second and third week), A-ROM exercises from the sixth session (third and fourth week), and strengthening exercises from the ninth session (fourth week; 10, 26, 27). The instruction sheet containing exercise explanations and the relevant figures will be given to the patients as they are expected to repeat the exercises 3 times a day with recommended dosages at home and the clinic [24]. Type of exercises are described in Table 2.

**Table 2:** List of therapeutic exercises

Type of exercise	Dose	Weeks
**Passive range of motion exercises:**		
Pendulum exercises Forward bow P-ROM exercises with physiotherapist for flexion, abduction, internal rotation and external rotation	3 times per day 3 sets X 10 repetitions	1st&2nd
**Active assisted range of motion exercises:**		
Wand exercises for flexion, internal rotation, extension and external rotation in supine position Wash the table (flexion on the table)	3 times per day 3 sets X 10 repetitions	2nd & 3rd
**Active range of motion exercises:**		
Pulley exercises in 3 directions including flexion, internal rotation and external rotation Wall slide Standing arm elevation in scapular plane Standing shoulder flexion	3 times per day 3 sets X 10 repetitions	3rd & 4th
**Strengthening exercises**		
Horizontal shoulder abduction and extension with low resistance trabands	3 times per day 3 sets X 10 repetitions	4th

#### Trigger point diagnosis

MTrP diagnosis will be conducted by a clinician (FN) with more than 4 years of experience in the management of MTrP using the following criteria: 1. Presence of a hyperirritable nodule in a palpable taut band of skeletal muscles, 2. Visible local twitch responses to the palpation of hypersensitive spots or taut bands, or 3. Reproduction of referred pain to a distant limb and causing distant motor and autonomic responses by palpating the sensitive spots appropriately [12]. These criteria have shown good inter-examiner reliability (κ, 0.84–0.88) when applied by an experienced clinician [25]. In case of palpating active MTrPs, the patients will report symptoms and their referred pain will be reproduced [12].

Participants will be examined for active MTrPs in the upper trapezius, lower trapezius, middle trapezius, supraspinatus, infraspinatus, subscapularis, teres minor, teres major, deltoid, levator scapulae, rhomboid major, and rhomboid minor muscles [26].

#### Experimental group

Prior to performing the dry needling procedure, patients should lie in a proper position and the exact place of the MTrP should be cleaned with a cotton soaked in alcohol. To perform the MTrP-DN, a 0.30 × 0.50 monofilament acupuncture needle (EACU™ Acupuncture Needles) will be used by the clinician. MTrPs will be detected through flat palpation of the hyperirritable nodule in a taut band that may produce referred pain or a local twitch response. The method of MTrP-DN will be based on the approaches presented by Dommerholt and Fernandez de-las-Penas. Therefore, the patients should be placed in a prone position to perform MTrP-DN for the following muscles: upper trapezius (posterior fibers), lower trapezius, middle trapezius, supraspinatus, infraspinatus, subscapularis (posterior fibers), teres minor, teres major, posterior deltoid, rhomboid minor, and rhomboid major muscles. The patients should lie supine for the following muscles: upper trapezius (anterior fibers), anterior deltoid, and subscapularis muscles (anterior fibers). Lastly, the patients should be in a side-lying position for the levator scapula, and middle deltoid [26]. The present study will apply the “fast in and fast out” technique which has been recommended by Hong. According to Hong’s technique, once the active MTrP is detected, the acupuncture needle will be inserted toward the MTrP until the first local twitch response is obtained. The needle will be inserted into and extracted from the muscle at approximately 1 Hz for 25 to 30 seconds [14, 27]. Finally, the needle will remain in the afflicted muscle for 20 minutes [28]*.* It should be noted that, MTrP-DN will only be used for a maximum of three muscles per session [14].

#### Control group

In the control group all the steps of applying dry needling including the position of the patient, size, and type of the needle will be similar to the experimental group. After placing the patient in the proper position and clearing the exact location of the MTrP, the needle will be inserted subcutaneously, by tapping (using the index finger) the needle into epidermis until it remains erect and can support its own weight. There will be no manipulation and twitch response and the needle will remain on the skin for 20 minutes [29, 30].

### Choice of comparators {6b}

Due to the necessity of combining therapeutic exercises with manual therapy and electrotherapy for an effective intervention, and to comply with the trial ethics for patients following RCR, the multimodal physiotherapy protocol based on the previous studies will be implemented for both the experimental and control group [10, 24, 31]. Moreover, according to the recent studies that have combined MTrP-DN with routine physiotherapy programs to investigate the effect of MTrP-DN in postoperative shoulder pain [14, 17], this study will use the real MTrP-DN for the experimental group and S-DN for the control group to facilitate participant blinding and control possible contamination bias. Penetrating S-DN technique demonstrated in the study of Braithwaite et al. as a participant blinding strategy will also be implemented in the current study [29, 30].

### Criteria for discontinuing or modifying allocated interventions {11b}

If a patient misses 2 sessions in a row or if they are not willing to continue their treatment for any reason, the intervention will be terminated and another patient will be replaced.

### Strategies to improve adherence to intervention {11c}

In order to encourage the patients, the entire rehabilitation protocol will be provided at no cost, and the patients will be closely in touch with their physiotherapist (FN) and surgeon (MNA and PT) to report their symptoms.

### Relevant concomitant care permitted or prohibited during the trial {11d}

Patients will be permitted to take analgesics like Tylenol in case of severe pain with the consultation of their surgeon.

### Outcomes {12}

#### Primary outcome measure

The main outcome measure for this study will be the resting pain measured by Numeric Pain Rating Scale (NPRS).

#### Key secondary outcome measure

This study’s key secondary outcome measure will be the glenohumeral active and passive ROM.

#### Secondary outcome measures

The secondary outcomes for this study will be the Shoulder Pain and Disability Index (SPDI), strength in shoulder movements.

### Participant timeline {13}

Recruitment of participants will be conducted by receiving a list of patients who have undergone RCR at Shafa Yahyaian Hospital and an interview with them by phone. According to the telephone interview, if the patients meet the study inclusion criteria, the necessary explanations will be given to them regarding the goals and treatment plan of the research study and after receiving their verbal consent for participation, they will be invited to the hospital for MTrP diagnosis and signing the informed consent form prior to baseline measurements and initiation of the treatment plan. The baseline measurements will be collected by forms from the patients which will contain the following descriptive characteristics: 1. age, 2. gender, 3. height, 4. weight, 5. medication history, 6. dominant hand, and 7. side of the operated shoulder. After completing the baseline measurements, the patients’ activity level will be collected using the Persian version of International Physical Activity Questionnaire (IPAQ [32];) Then, the randomization process will be performed using the block-balanced randomization method and the patients will be randomly assigned into an experimental or control group. Following the randomization process, outcome measure assessments will be conducted by a blind assessor before the start of the first treatment session and after the end of the tenth session (2 times of evaluation in total). Participants will receive 10 treatment sessions over 4 weeks (3 sessions a week) and 4 sessions of MTrP-DN or S-DN (once a week). The study’s schedule is depicted in Table 3.

**Table 3:**
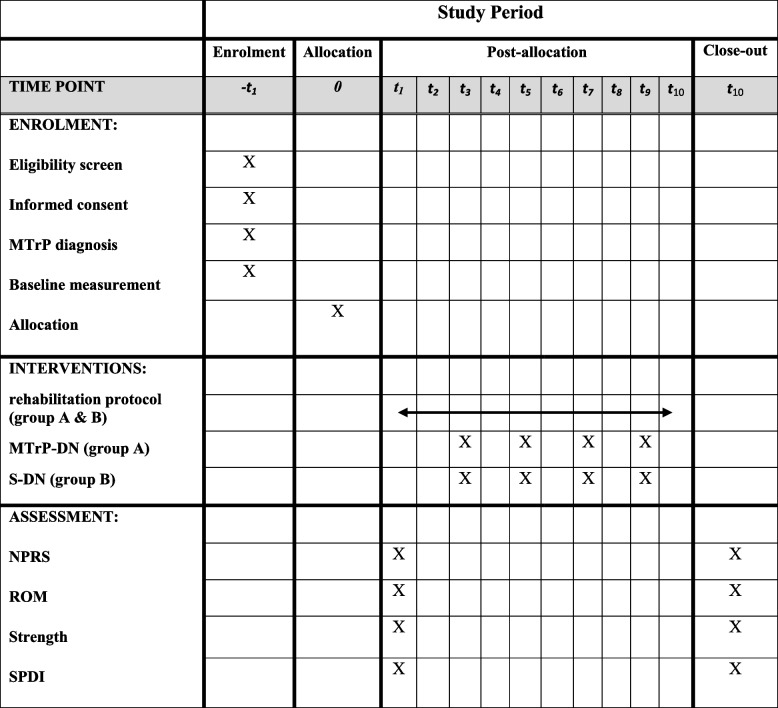
Study’s time schedule

### Sample size {14}

G-Power software (version 3.1.9.4) was used in order to determine the sample size. F-tests as a family test and Analysis of Covariance (ANCOVA) as a statistical test were utilized. According to Michener et al., Minimal Clinically Important Difference (MCID) in post-operative shoulder pain measured by NPRS was reported to be 2.17 [33]. The effect size was calculated to be 0.66 based on a prior study conducted by Halle et al. in NPRS after DN following shoulder stabilization repair [17]. Power and α error were set at 95% and 0.05, respectively. Required sample size was estimated at 34 prior to commencement of the trial. To account for a potential 30% of attrition rate, 46 subjects will be recruited.

### Recruitment {15}

The participants will be recruited based on the list of patients who have undergone RCR in Shafa Yahyaian Hospital and interviewing them by telephone**.**

### Sequence generation {16a}

All eligible RCR participants will be randomly assigned into either MTrP-DN combined with multimodal rehabilitation protocol (experimental group) or S-DN combined with multimodal rehabilitation protocol (control group) with an allocation ratio of 1:1. Randomization will be performed using variable blocks with 4 character-blocks containing letters A or B (letter A indicates ‘experimental group’ and letter B indicates ‘control group’). After randomizing, the randomization schedule will be transferred into written instructions and will be placed in sequentially numbered, opaque, and sealed envelopes. The randomization process will be performed by someone who is outside the research team before the beginning of the study.

### Allocation concealment mechanism {16b}

Following the initial evaluations, the numbered envelopes will be given to each patient according to the ordinal number of each person admitted to the study. Once each patient enters the first treatment session, the therapist will plan the treatment intervention based on the information included in their envelope. After placing the patients in the target group, they are asked not to provide their allocation information to the examiner to prevent data contamination.

### Implementation {16c}

The allocation sequence, enrolling, and assigning participants to interventions will be performed by the clinic’s secretary.

### Blinding {17a}

This is a double-blinded study. The patients and the outcome assessor will be informed about the treatment process but they will be blinded regarding the allocation during the study.

### When unblinding is permissible {17b}

There will be no permission for unblinding the outcome assessor or the patients during the study, but the patients will be informed about their group allocation after the study is finished.

### Data collection methods {18a}

Assessments will be performed by the fifth author (SA).

#### Primary outcome measure

##### Resting pain

NPRS will be used to assess the resting pain. This scale is scored from 0 to 10 and the patients will be asked to report their level of pain in the past 24 hours with the instruction that “0 is no pain”, and “10 is the worst pain imaginable”. The NPRS is a valid and reliable tool for patients with shoulder pain [34, 35].

#### Key secondary outcome measure

##### Active and passive ROM

ROM will be measured using a standard 18 cm plastic goniometer. The following shoulder movements will be measured: flexion, abduction, external rotation and internal rotation to the non-painful end range [36]. Glenohumeral flexion will be performed with the patient in crook-lying position and arms at the side. The assessor will grasp distal of the humerus and apply glenohumeral flexion to the limit of non-painful motion. The stationary arm of the goniometer will be placed parallel to the lateral midline of the trunk. The movable arm of the goniometer will be placed parallel to the longitudinal axis of the humerus. Glenohumeral abduction will be performed with the patient in supine and arms at the side. The assessor will grasp distal of the humerus and move the humerus through non-painful abduction. The stationary arm of the goniometer will be placed parallel to the sternum and the movable arm will be placed parallel to the longitudinal axis of the humerus. Glenohumeral external rotation will be performed in supine position, shoulder in 90° of abduction, elbow in 90° of flexion and the forearm in mid-position. The assessor will then move the shoulder to the limit of non-painful external rotation. The stationary arm of the goniometer will be placed perpendicular to the trunk and the movable arm will be placed parallel to the longitudinal axis of the ulna. Glenohumeral internal rotation will be performed in prone position, shoulder in 90° of abduction, elbow in 90° of flexion and forearm in mid-position. The assessor will move the shoulder to the limit of non-painful internal rotation. To measure the glenohumeral A-ROM, the patient’s start position and the goniometer placement will be the same as the P-ROM methods. The patient will be asked to perform the relevant movement actively through non-painful ROM and the assessor will prevent substitute movements and perform the measurement.

#### Secondary outcome measure

##### Shoulder pain and disability index (SPDI) Persian version

This index will be used to evaluate the functional status of the patients regarding their shoulder pain and disability. SPDI is a self-administered 13-item questionnaire. It consists of 2 subscales: a 5-item subscale assessing the patient’s pain level and an 8-item subscale assessing the patient’s shoulder disability in activities of daily living. Patients will be instructed to choose the number that best describes their level of pain and disability [37]. The Persian version of the SPDI has proved to be a reliable and valid instrument to be implemented for the Persian-speaking population with shoulder disorders (Cronbach’s α = 0.94 and intraclass correlation coefficient = 0.84; [38]).

##### Strength in shoulder movements

Strength will be measured with a handheld dynamometer (SF-50 Digital Force Gauge Dynamometer). The measurement will be recorded during flexion and abduction in kilogram-force (kgf). Each movement will be repeated 3 times and the patient will hold each isometric contraction for 5 seconds and the average of these 3 trials will be calculated to be used in data analysis. Flexion isometric force will be performed with the patient in short sitting and arms at the side. The assessor will stand on the test side and ask the patient to raise the arm forward to 45° flexion while extending the elbow and pronating the forearm. The assessor will place the handheld dynamometer over the distal of the humerus just above the elbow and her other hand will stabilize the shoulder. Resistance will be applied downward and the patient will hold the isometric contraction for 5 seconds. Measurement of abduction isometric force will be taken with the patient in sitting position and arms at the side. The assessor will stand behind the patient and ask the patient to elevate the arm to the side with the shoulder in 45° abduction, forearm in neutral position and the thumb pointing up. The assessor will place the handheld dynamometer over the arm, just above the elbow and stabilize the shoulder with the other hand. Resistance will be applied downward and the patient will hold the isometric contraction [23, 39].

### Adverse events

The items reported for adverse events by Boyce et al. will be used in this study as well. In case of any adverse events depicted in Table 4, the physiotherapist will report them [40].

**Table 4:** Adverse events reported with dry needling

Event	Number reported	Percentage per total treatments
Bleeding		
Bruising		
Pain during		
Pain after		
Aggravated symptoms		
Drowsiness		
Feeling faint		
Headache		
Nausea		

### Plans to promote participants’ retention and complete follow-up {18b}

During the study’s four weeks of the treatment plan, the patients are encouraged to regularly contact the physiotherapist (FN) and the surgeons (MNA or PT). The physiotherapist will also call the patients once a week to check on their home exercises and ask about their problems. Additionally, there won’t be any costs associated with the entire treatment to motivate the patients further.

### Data management {19}

Each patient’s information including baseline measurements, personal information and outcome measures will be recorded on prefabricated forms by the blind assessor (SA). After data collection on prefabricated sheets, the information will be stored confidentially on the assessor’s laptop and all outcome measures’ data will be recorded in an Excel file for analysis.

### Statistical methods for primary and secondary outcomes {20a}

Data will be analyzed using STATA software version 16 (StataCorp LLC). In order to investigate the normal distribution of data, the Shapiro-Wilk test, mean and median similarity, analysis of histogram, and the skewness and kurtosis analysis of the data distribution will be used. In case of data’s normal distribution, parametric tests will be used to analyze the data before and after intervention. If the homogeneity of data is confirmed by Levene’s test, (ANCOVA) will be performed. In this test, the covariance variable will be the initial data value before the intervention. The significance level of the tests will be defined as 0.05. In addition to the significance index, the effect size index will be analyzed by Cohen’s d method to compare the two treatment methods for each variable to determine the effect of the intervention on each dependent variable, regardless of the sample size. According to the newly presented definition of effect size it is divided as follows: from 0.01 to 0.2: very small, from 0.2 to 0.5: small, from 0.5 to 0: medium, from 0.8 to 1.2: large, from 1.2 to 2 very large, more than 2: huge [41].

### Methods for additional analyses (e.g. subgroup analyses) {20b}

There will be no plan for additional analysis in this study.

### Methods in analyses to handle protocol non-adherence and say statistical methods to handle missing data {20c}

Protocol non-adherence or lost to follow-up will be handled by intention to treat analysis.

### Data monitoring {21a}

This study will be conducted under the supervision of the faculty members of the Rehabilitation Research Center, Department of Physiotherapy, School of Rehabilitation Sciences, Iran University of Medical Sciences, Tehran, Iran. In addition, the Data Monitoring Committee (DMC) has no competing interest in any stages of this study and is completely independent from the sponsor.

### Interim analyses {21b}

There will be no interim analysis in this study.

### Harms {22}

The patients will be informed about the possible adverse events following MTrP-DN prior to signing the consent form. Also, occurrence of any adverse events following MTrP-DN will be managed by the expert physiotherapist (FN) and recorded as the adverse events of the study. If the patients are not willing to continue the trial for the adverse events or any other reason, they can leave the study.

### Auditing {23}

There will be no auditing trial in this study.

### Protocol amendments {25}

In case of important protocol modifications, protocol amendments will be updated on https://www.irct.ir/.

### Consent or assent {26a}

The informed consent form will be prepared according to the standards of the Ethics Committee of Iran University of Medical Sciences and will be obtained from the patients by the hospital’s secretary. All eligible patients will be informed about the interventions and the adverse events prior to signing the consent form. In the Appendix section, you can find more information about the consent form.

### {26b}

Ancillary studies are not applicable in the current study.

### Confidentiality {27}

All patients’ data will be archived in a unique confidential manner at all stages of the trial and this confidentiality will be protected during and after the trial.

### Ancillary and post-trial care {30}

For those patients who experience major adverse events following MTrP-DN or any other harms caused due to participating in the trial, the research team will compensate for all the occurred harms throughout the trial.

### Trial results {31a}

This study aims to publish the results in an international peer-reviewed journal and also present the results in a recognized national or international conferences.

### Authorship {31b}

In the present study, the Vancouver guideline was implemented for determining the authorship eligibility.

### Reproducible research {31c}

For granting public access to the full protocol, participant-level dataset and statistical code, sending a reasonable email to the corresponding author will help an eligible investigator to receive the required information.

## Discussion

Postoperative shoulder pain is a common complaint in patients after RCR [14]. The procedure of surgery as a mechanical trauma can be a potential mechanism for activation of MTrPs [12]. A study by Torres-Chica et al. has suggested that surgical procedure may change latent MTrPs to active MTrPs by an increase in the release of inflammatory mediators [42]. Consequently, adding a muscle treatment approach to the rehabilitation program after RCR surgery can be beneficial for the patients.

Since exercise therapy after RCR surgery is an essential part of a physiotherapy treatment plan, an appropriate progressive exercise program will be included in this protocol to be followed during the treatment and also at home [24]. Exercises in this protocol include progressive ROM and strengthening exercises with accurate timing of each stage to minimize retearing of tendons and optimize the outcomes [10].

The efficacy of combining manual therapy, exercise therapy, and electrotherapy has been proved in previous studies for patients with rotator cuff disease [11, 43]. Therefore, this protocol contains different manual therapy techniques, exercise therapy, and electrotherapy programs. Furthermore, due to the presence of MTrPs in patients with postoperative shoulder pain [12], MTrP-DN may help to facilitate the recovery following RCR. While various studies have provided evidence for using dry needling for patients with myofascial pain syndrome of the neck and upper extremity [16], including a single session of dry needling in a multimodal physiotherapy program showed to help the patients recover their function faster in the condition of postoperative shoulder pain [14]. Therefore, this trial will include 4 sessions of MTrP-DN in a multimodal rehabilitation protocol to investigate the effectiveness of MTrP-DN on postoperative shoulder pain after RCR.

Due to the lack of similar studies for the management and rehabilitation following RCR surgery, the implementation and results of this study will make a new contribution to the rehabilitation of patients with postoperative shoulder pain and dysfunction, and it may also help to establish a new line of research for adding MTrP-DN in rehabilitation protocols following RCR surgery.

### Trial status

This study protocol is the first version, finalized in October 2022, and is recruiting participants. The recruitment process is expected to take 5 months.

## Data Availability

The final trial dataset will be accessible by sending a justifiable email to the corresponding author (MD).

## Appendix

### Informed consent materials {32}

The consent form items have been designed for Persian-speaking participants based on the suggestions provided on the website of the Ethics Committee of Iran University of Medical Sciences.

## Abbreviations

MTrP: Myofascial trigger point
MTrP-DN: Myofascial trigger point dry needling
S-DN: Sham dry needling
NPRS: Numeric Pain Rating Scale
SPDI: Shoulder Pain and Disability Index
ROM: Range of motion
A-ROM: Active range of motion
P-ROM: Passive range of motion
AA-ROM: Active assisted range of motion
RCT: Rotator cuff tear
RCR: Rotator cuff repair
ANOVA: Analysis of covariance
MCID: Minimal Clinically Important Difference
IPAQ: International Physical Activity Questionnaire
